# Development of Surface-Enhanced Raman Scattering (SERS)-Based Surface-Corrugated Nanopillars for Biomolecular Detection of Colorectal Cancer

**DOI:** 10.3390/bios10110163

**Published:** 2020-10-31

**Authors:** Kuan-Hung Chen, Meng-Ju Pan, Zoljargal Jargalsaikhan, Tseren-Onolt Ishdorj, Fan-Gang Tseng

**Affiliations:** 1 Institute of NanoEngineering and MicroSystems, National Tsing Hua University, HsinChu 30013, Taiwan; s102035808@m102.nthu.edu.tw; 2 Engineering and System Science Dept., National Tsing Hua University, HsinChu 30013, Taiwan; evq50222@gmail.com; 3 School of Information and Communication Technology, Mongolian University of Science and Technology, Ulaanbaatar 13341-0048, Mongolia; j.zoljargal@must.edu.mn (Z.J.); tseren-onolt@must.edu.mn (T.-O.I.); 4 Frontier Research Center on Fundamental and Applied Sciences of Matters, National Tsing Hua University, HsinChu 30013, Taiwan; 5 Research Center for Applied Sciences, Academia Sinica, Taipei 11529, Taiwan

**Keywords:** high-aspect-ratio nanostructures, corrugated nanopillars, SERS, nanobiosensor, miRNA detection

## Abstract

In this paper, a nanobiosensor with surface-enhanced Raman scattering (SERS) capability is introduced for highly sensitive miRNA detection in colorectal cancer. This sensor was designed and fabricated by employing a nanoshielding mechanism from nanopolystyrene beads to resist reactive ion etching and allow anisotropic electrochemical etching, producing high-aspect-ratio, surface-corrugated nanopillars (SiNPs) on a silicon wafer to create extensive hot spots along the nanopillars for improved SERS signals. SERS enhancements were correlated with nanorange roughness, indicating that hot spots along the pillars were the crucial factor to improve the SERS effect. We achieved the detection capability of a trace amount of R6G (10^−8^ M), and the SERS signal enhancement factor (EF) was close to 1.0 × 10^7^ on surface-corrugated gold SiNPs. miRNA samples were also demonstrated on this sensor with good sensitivity and specificity. The target molecule miR-21-Cy5 was easily monitored through Raman spectrum variation with a PCR-comparable concentration at around 100 pM with clear nucleotide-specific Raman signals, which is also suitable for biomolecule sensing.

## 1. Introduction

Colorectal cancer (CRC) is a prevalent type of cancer that varies widely around the world [1,2]. Among the various cancers, CRC has been a leading cause of morbidity and mortality over the past years [3]. The recovery rate is high following surgery treatment in the early stages, but it dramatically decreases in the third and fourth stages. Therefore, early diagnosis and treatment are crucial to increase survival rates among CRC patients [4,5]. The current method of CRC early stage diagnosis relies on symptoms, colonoscopy screening, or fecal occult blood testing (FOBT) in the majority of patients. However, CRC symptoms are similar to gastrointestinal disorders, and the sampling is invasive and inconvenient, which reduces suspected patients’ motivation. Moreover, the false positive rate of FOBT is nearly one in four [6], thus requiring further diagnosis. Furthermore, CRC is inhomogeneous and has complex gene expression subtypes [7]. Medical professionals have considered molecular subtypes of CRC for personalized medicine and optimal treatments [8]. As a result, scientists can provide further insight in terms of molecular information for developing new diagnostic approaches.

MicroRNAs (miRNAs), which are small noncoding RNA sequences containing 18–24 nucleotides, are transported by exosomes into bodily fluids as post-transcriptional regulators of gene expression. There is a growing body of evidence showing that exosomal miRNAs have a strong correlation with the occurrence and development of various types of tumors [9,10,11,12]. Those findings have attracted increased attention to miRNAs as ideal markers for early diagnosis and prognosis, particularly in CRC [13,14]. For example, microRNA-21 (miR-21) in serum was found to be highly associated with early stages of CRC [15]. Moreover, several studies have shown an association between elevated levels of miR21 and the downregulation of tumor suppressor genes [16,17,18,19,20]. Multiple methods have been developed for miRNA detection, including polymerase chain reaction (PCR) [21] and Northern blotting [22]. However, these methods are laborious and tedious, and so far, the detection of exosomal miRNA molecules is still considered to be challenging work.

Surface-enhanced Raman scattering (SERS) spectroscopy is a powerful analytical tool for miRNA detection [23,24,25,26,27,28]. SERS is carried out when nanostructured surfaces of noble metals (usually gold or silver) absorb irradiation with light; the energies are transferred to localized surface plasmon resonance (LSPR) with near substrates, and ordered signals are enhanced. The crucial key is the nanogaps among the metals generating strong SERS effects, known as “hot spots”. For example, individual metal nanoparticles have SERS enhancement factors (EFs) of 10^3^ [29], but the aggregated nanoparticle Au/Ag alloy model with nanogaps can reach EFs of 10^8^–10^10^. Other nanostructures with sub-10 nm edges or gaps, such as nanoflowers [30] or nanostars [31], have been reported to promote EFs of 10^7^–10^8^. However, the inhomogeneous aggregation of nanoparticles or the imbalanced distribution of hot spots may result in irreproducible measurements. For instance, in the AuNP colloidal system, the Raman intensities dropped 1000-fold when the spacing expanded 2-fold from the narrowest 2–4 nm [32].

To address this problem, modified ordered nanostructures on solid surfaces by mature lithography fabrications have been developed, such as nanobowls [26], nanoholes [33], and nanopillars (nanowire or nanorod arrays) [33,34,35,36,37,38]. The gaps between these adjacent nanostructures produce SERS enhancement due to strong electromagnetic fields [39,40] and plasmon coupling effect [41]. Huang et al. demonstrated that silicon nanopillar (SiNP) arrays produce a reproducible SERS signal of long-sequence DNA with a small error bar [38]. They demonstrated that the open spaces between pillars expand the detection of DNA length (25–50 nm in length). However, SERS enhancement of original SiNPs was around ~10^3^, which was not strong enough to reach the goals of label-free or labeled RNA detection at the ~pM level. The reason for this was that the hot spot numbers were not sufficient and efficient enough to obtain a strong SERS effect. Several studies [36,37] decorated silver or gold nanoparticles on SiNPs to improve the EF to 10^6^–10^9^, but the uniformity of decoration was a challenge, which may hinder stability or mass production. Other studies formed these ordered nanorods by electron beam lithography (EBL) and the oblique angle vapor deposition (OAD) method to build up condensed silver nanorod arrays that provided EFs up to 10^8^ [34,35]_._ In our previous studies, we found that SERS enhancements on etched nanospheres were dominated by sub-10 nm pitches and high surface roughness, instead of particle size [42]. The surface roughness and sub-10 nm nanocorrugations on polystyrene beads were also quantified to provide 2 orders of magnitude of SERS enhancements [42]. As a result, in the current study, not only the aspect ratio of nanostructures but also the surface roughness of SiNPs were adjusted to further improve the SERS efficiencies and signals.

**Figure 1:**
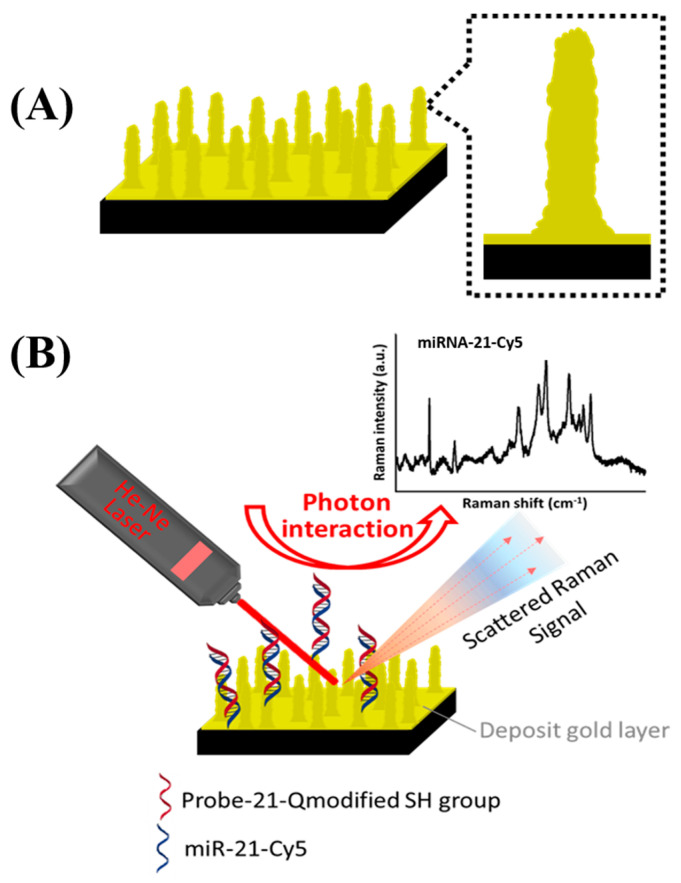
(**A**) Schematic of corrugated nanopillar array with rough surfaces. (**B**) The sensing scenario of target microRNA-21 (miR-21) on this SERS enhancement substrate.

In this study, we not only demonstrated the fabrication process of silicon nanopillars with the nanoshielding mechanism, but we also increased the surface roughness to markedly improve the Raman signals. Not having secondary modifications and the chemical synthesis of nanoparticles may reduce complicated procedures and provide benefits for mass production. We further combined SERS-active SiNPs with sensitive molecular probes, which can immobilize onto the pillar surfaces to obtain a fingerprint spectrum for miR21 recognition and detection, as shown in Figure 1. These characteristics are beneficial for highly sensitive and stable detection of RNA molecules.

## 2. Materials and Methods

### 2.1. Materials

Polystyrene beads (PSBs) with a diameter of 530 nm were purchased from Thermo Fisher Scientific, Waltham, MA, USA. For chemical etching, 30% hydrogen peroxide was purchased from Acros Organics, Geel, Belgium. Hydrofluoric acid (HF), rhodamine 6G, tris(2-carboxyethyl) phosphine hydrochloride (TCEP), and phosphate-buffered saline (PBS) were purchased from Sigma-Aldrich, St. Louis, MO, USA.

### 2.2. Oligonucleotides

To recognize and quantify the amount of target miRNA molecules, we employed a fluorescence-quenching/SERS-enhancement design to test miR21. A typical miRNA biomarker for CRC, miR-21: 3′-Cy5-T AGC TTA TCA GAC TGA TGT TGA-5′ [43], was modified with Cy5 (by Integrated DNA Technologies, Inc. (IDT), USA) and defined as miR-21-Cy5. The attached fluorescence dye, Cy5, was used for molecule quantification and also as a Raman tag to enhance the Raman signal of miR-21 [44]. The complementary sequence 3′-SH-isp18-TCA ACA TCA GTC TGA TAA GCT A-Quencher-5′ was defined as Probe-21-Q. The sequences were a modified 18-atom hexa-ethyleneglycol spacer (isp-18), and the quencher was Iowa Black® FQ, a patented molecule from IDT, which has a broad absorbance spectrum ranging from 420 to 620 nm for Cy5 fluorescence quenching. When the target miR-21-Cy5 molecules were hybridized onto Probe-21-Q molecules immobilized on the sensor surface, the Cy5 fluorescence was quenched to reduce the fluorescence signals in the solution. At the same time, the Raman signature spectra of the nucleotides were observed through the enhancement of high-aspect-ratio SiNPs. All sequences are listed in Supplementary Table S1.

### 2.3. Fabrication of Nanopillar Structures by Nanoshielding Methods

**Figure 2:**
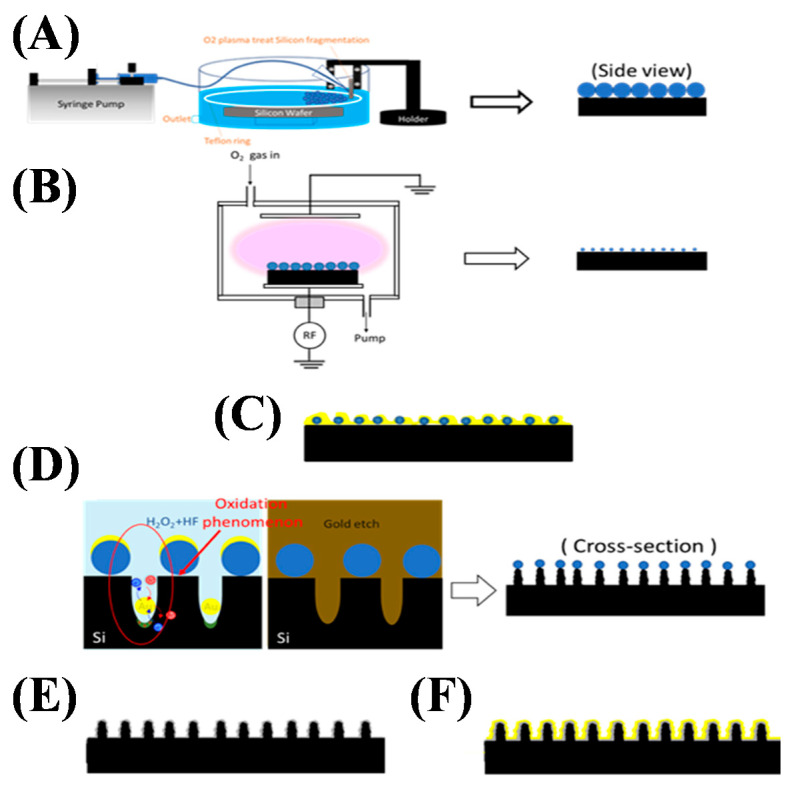
Fabrication process of the corrugated nanopillar structures. (**A**) Self-assembled 530 nm Polystyrene beads (PSBs) on silicon substrate. (**B**) O_2_ reactive ion etching (RIE) treatment to shrink PSBs. (**C**) First E-gun deposition of 5 nm Au as a catalyst for Si and H_2_O_2_ to form SiO_2_. (**D**) Chemical etching by HF:H_2_O_2_ = 9:1 and removal of residual Au by gold etching. (**E**) Dry oxidation on silicon surface to form 100 nm SiO_2_ for insulation. (**F**) Second E-gun deposition of 5 nm gold for SERS.

The fabrication process of the nanopillar array is illustrated in Figure 2. Firstly, to assemble a single layer of uniform and close-backed beads, 530 nm PSBs were mixed with alcohol, and water was carefully flowed on the surface. Subsequently, the water was slowly drained, and the PSBs were gradually laid on a silicon wafer surface using surface tension and self-assembled into a monolayer. The PSB-coated wafers were then cut into 2 mm square pieces as SERS substrates for further experiments. O_2_ reactive ion etching (RIE) treatment (with a flow rate of 10 sccm, RF power of 200 W, and process time ranging from 40 to 160 s with a time interval of 40 s) was then employed to shrink the size of the PSBs to the designated values. These PSB arrays were utilized as the nanomasks for the electrochemical etching process to form corrugated nanopillar arrays, as shown in Figure 2d,e. 

Successively, a layer of 20 nm gold film was deposited on the silicon substrate as a catalyst for Si reacting with H_2_O_2_ to form SiO_2_ [45,46]. Then, the substrate was immersed into a chemical etching solution (HF:H_2_O_2_ = 9:1) for the electrochemical reaction process. In this process, two reactions were performed simultaneously. Firstly, an electrochemical reaction that induced hole generation was carried out at the interface between H_2_O_2_ and the metal. The holes then traveled through the gold clusters, and the Si substrate was subsequently oxidized locally beneath the gold clusters to form SiO_2_. Secondly, a chemical complexation reaction dissolved silicon oxide via HF (i.e., the removal of SiO_2_). Since the oxidation mainly took place underneath the metal clusters, the chemical etching process on the silicon substrate was anisotropic, so high-aspect-ratio nanopillar arrays were obtained. This was followed by dry oxidation of the silicon surface to generate 100 nm SiO_2_ for surface coverage to prevent the connection among pillars to maintain SERS efficiency. Finally, a second gold-layer deposition (thickness of 5 nm at a deposition rate of 1.0 Å/s) was performed as the source of plasmon resonance to enhance signals. The deposited 5 nm of gold material formed clusters on the pillar surfaces, providing strong electromagnetic coupling in our experiments without bridging the sub-10 nm gaps among the pillars, providing numerous SERS hot spots along the nanopillar surfaces.

### 2.4. Probe Preparation and Immbolization

Probe-21-Q conjugated on the nanopillar surface by strong interaction between the thiol group and the gold [45]. In order to activate the thiol group on the 5′ terminal of Probe-21-Q, 25 μL of TCEP with a concentration of 10 μM was mixed with 25 μL of Probe-21-Q at a concentration of 1 μM in PBS buffer at 24 °C for 30 min. Thiol-activated Probe-21-Q was immobilized on chips overnight.

### 2.5. Characterization of Nanopillar Chips with Scanning Electron Microscopy and Atomic Force Microscopy

**Figure 3:**
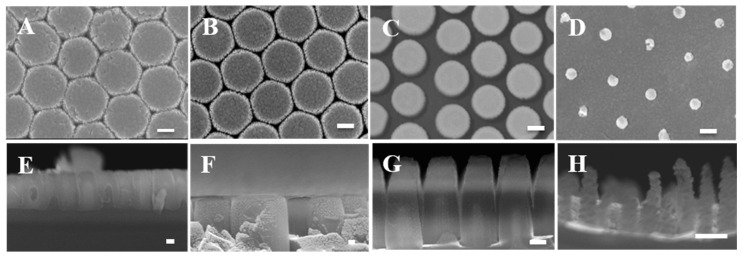
SEM images of SiNP arrays with different RIE etching times. (**A**–**D**) show the top views and (**E**–**H**) show the cross-section views of nanopillars fabricated by RIE at 40, 80, 120, and 160 s of etching and the later electrochemical wet etching process, respectively. The scale bar represents 100 nm.

A high-resolution thermal field emission scanning electron microscope (HRFEG-SEM) (JSM-7610F, JEOL, Tokyo, Japan) was employed to image the structures of the corrugated nanopillar arrays fabricated under different etching conditions, with a top view at 50 kV and a cross-sectional view at 15 kV. The SEM results are shown in Figure 3.

The surface roughness was further investigated with atomic force microscopy (AFM, JPK NanoWizard, Neuchatel, Germany). The AFM was operated in tapping mode with a 512 × 512 pixel resolution.

**Figure 4:**
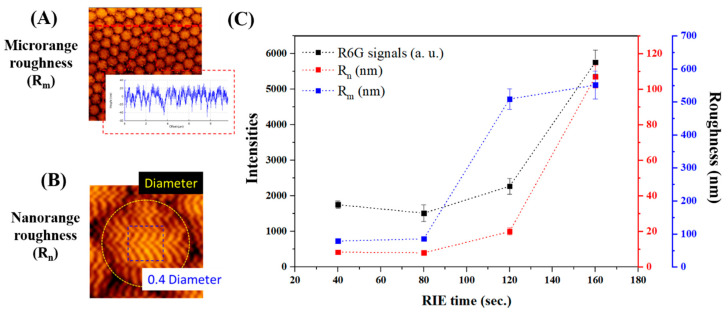
Corrugated nanopillar structures for SERS enhancement. (**A**) Atomic force microscopy (AFM) analysis on a large area covered by hundreds of corrugated silicon nanopillars (SiNPs) for microrange roughness (R_m_). (**B**) AFM analysis of nanostructures on the top region of one corrugated nanopillar for nanorange roughness (R_n_). (**C**) Relationship between Raman intensities (black curve), R_m_ (blue curve), and R_n_ (red curve).

The nanocorrugation-contributed roughness (NCR) is described as the average roughness (R_a_), the square roughness (R_q_), and the peak-to-valley roughness (R_v_) obtained with the JPK Data Processing software. To understand the contributions of SERS enhancements, we independently analyzed microranges and nanoranges of roughness of the SiNPs. For analyzing microrange roughness (R_m_), we calculated R_v_ values from 9 μm long cross-sections of chips. R_v_ represents the averaged valley depth below the mean line, which was mostly contributed by the pillar’s morphologies as shown in Figure 4A. On the other hand, we defined R_n_ by analyzing the average roughness (R_a_) from a selected 0.4 square area on the top of the pillars to describe the nanostructures among the SiNPs as shown in Figure 4B. AFM data were obtained by averaging the roughness values of six different samples. To estimate the relationship between SERS signals and roughness, Pearson’s correlation coefficients (Pearson’s *r*) were analyzed in Figure 4C.

### 2.6. SERS Measurements

**Figure 5:**
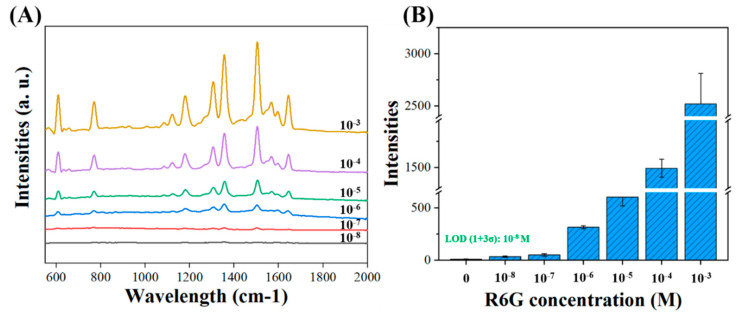
(**A**) Raman spectra of series concentrations of R6G (10^−3^–10^−8^) on SiNPs (RIE 160 s). (**B**) Relative Raman intensities at a wavelength of 1360 cm^−1^ at series concentrations of R6G.

SERS spectra were acquired by a micro-Raman spectrometer (LabRAM HR, HORIBA Jobin Yvon, France) using a 632.8 nm He–Ne laser. The laser output power was around 3 mW. A 50× objective (MPLAN 50×/0.75, OLYMPUS) was utilized to focus on the nanopillars, and the laser spot size was adjusted to 2.86 μm. The exposure time for the SERS measurement was set to 10 s, with a 632 nm notch filter to filter out Rayleigh scattering signals. Two microliters of rhodamine 6G (R6G) with a concentration of 2 mM were placed on different nanopillars (see Figure S2). Raw SERS spectra were processed through LabSpec application software for smoothing the data and adjusting the baseline before further analysis. Furthermore, to analyze the detection limits, series concentrations of R6G (from 10^−3^ to 10^−8^ M) were placed on the SiNPs with RIE-160 s, and Raman signals were recorded (Figure 5). Distilled water was utilized for recording blank signals. In the SERS comparison, we evaluated the relative Raman intensity, which was quantified with the maximum peak intensity at 1356 cm^−1^, subtracting the baseline intensity at 1356 + 20 cm^−1^ (see Figure 5B). The limit of detection (LOD) of R6G was estimated by comparing the lowest concentration with the blank signals adding 3 times of deviation.

**Figure 6:**
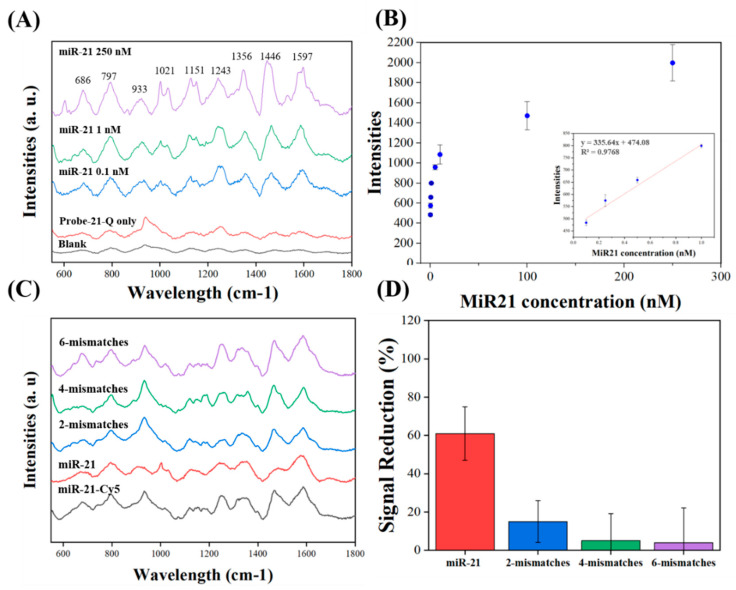
Raman spectra of miR-21 detection with SiNPs. (**A**) Full fingerprint spectrum. From bottom to top: PBS buffers, probe only, and probe hybridized with miR-21-Cy5 at concentrations of 0.1, 1, and 250 nM marked with recognized peaks. (**B**) Point data versus series concentrations (0.1-250 nM) of miR-21-Cy5. The inserts show a linear relationship from 0.1 to 10 nM. (**C**) Raman spectrum and (**D**) reduced Raman signals at 1466 cm^−1^ after competitive testing with miR-21, miR-21-mis2, miR-21-mis4, and miR-21-mis6.

For miR-21 detection, SERS spectra were acquired by a portable Raman spectrometer (RAPIS-785, Phansco, Taiwan) using a 785 nm near-infrared (NIR) diode laser. Samples were excited using 30 mW laser power. The exposure time for the SERS measurement was set for 1 s. miR-21-Cy5 was diluted with PBS buffers at concentrations from 0.1 to 250 nM and then immobilized with chips for 1 h. After washing two times, the SERS spectra were recorded by the application software. We evaluated the relative Raman intensity, which was quantified with the maximum peak intensity at 1446 cm^−1^, subtracting the baseline intensity at 1530 cm^−1^ (see Figure 6B). The accuracy was represented by the relative standard deviation (RSD).

To test the specificity of this sensor, miR-21 similar sequences with 2, 4, and 6 mismatched bases were used to compete with Probe-21-Q’s binding sites. After 100 nM of miR-21-Cy5 and Probe-21-Q hybridization as described in the previous section, the complementary sequences (miR21, miR-21-mis2, miR-21-mis4, and miR-21-mis6) under the same concentration were added to compete with the sequences for 1 h. The sensors were washed two times with PBS buffer to remove nonbinding sequences. miR-21-Cy5 signals were measured and recorded as described. The specificity analysis is also illustrated in miR-21 solutions with fluorescent detections, as shown in Figure S4.

## 3. Results

### 3.1. Fabrication of Nanopillar Biosensors

SiNPs morphologies were analyzed by SEM, and microrange and nanorange roughness by AFM. Figure 3 shows the top view and cross-section view of SiNPs by SEM. For SiNPs after applying 40, 80, 120, and 160 s of RIE treatments and chemical etching, the average diameters of the pillars were 530, 520, 430, and 150 nm, respectively. From the cross-sectional view, the pillar heights and aspect ratios were 980, 1130, and 530 nm and 1.88, 2.63, and 3.53 for 80, 120, and 160 s, respectively. Note that pillar structures did not form within etching times shorter than 40 s in our experiments. After optimization, the structures with 160 s etching time provided a more uniform and repeatable nanopillar structure, with an average top width of 96 nm, height of 486 nm, aspect ratio of 5.06, and areal density of 5 pillars/μm^2^. Moreover, the surface roughness of microrange structures (R_m_) and nanorange structures (R_n_) on the pillars’ heads was measured by AFM, as shown in Figure 4. In micro- or nanorange measurements, NCR parameters had the same trends with RIE time (shown in Figure S1). For SiNPs with RIE−40 s, −80 s, −120 s, and −160 s, R_m_ was 78.6 ± 3.8, 85.9 ± 5.7, 508.7 ± 31.2, and 551.2 ± 42.2; and R_n_ was 7.8 ± 1.1, 8.4 ± 0.7, 20.0 ± 1.5, and 106.8 ± 11.0, respectively. These findings align with the morphologies in the SEM images. Furthermore, SERS signals were measured from five fabricated samples. We found the relationship between R_m_ and R_n_ with Pearson’s correlation. Pearson’s correlation coefficients of SERS signals with R_m_ were 0.811, and with R_n_ were 0.998.

### 3.2. SERS Signal Enhancement by Corrugated Nanopillar Biosensor

To detect the SERS enhancement on the series of nanopillar structures, we compared the Raman intensity of rhodamine 6G (R6G) dye (Figure S2). Typical Raman spectra of R6G were well resolved with the following peaks: 612 cm^−1^ (C–C–C in-plane bending); 775 cm^−1^ (C–H out-of-plane bending); 1189 cm^−1^ (C–H in-plane bending); 1312 cm^−1^ (C–O–C stretching); and C–C stretching modes for aromatic rings at 1356, 1509, 1576, and 1651 cm^−1^. We further calibrated series concentrations of R6G from 10^−3^ to 10^−8^ M with SiNPs with RIE-160 s. The SERS signals were measured from five of the surfaces at 160 s, as shown in Figure 5 The limit of detection (LOD) of R6G was estimated to be 10^−8^ M based on three time measurements of blank signals. The enhancement factor (EF) was calculated with the following equation:

$$\mathrm{EF}=\frac{I_{\mathrm{SERS}}}{I_{\mathrm{NRS}}}\frac{/C_{\mathrm{SERS}}}{/C_{\mathrm{NRS}}}$$

where I_SERS_ and I_NRS_ are the intensities of the characteristic absorption peak in SERS and the normal Raman spectrum, respectively, and C_SERS_ and C_NRS_ are the concentrations utilized for SERS (I_SERS_) and normal Raman spectrum intensities (I_NRS_), respectively. In our experiment, I_NRS_ was obtained from 0.01 M of R6G solution with bare silicon. Its average intensity was 3.5 counts. C_SERS_ was 10 nM. The EF of this SiNP was obtained close to 1.0 × 10^7^.

### 3.3. miR-21 Detection on Corrugated Nanopillar Biosensors

The hybridization process between the target molecule miR-21-Cy5 and Probe-21-Q could easily be distinguished through Raman spectrum variations, as shown in Figure 6. Without trapping miR-21-Cy5, the Raman signal for Probe-21-Q conjugated on the gold surface represented a small broadband spectrum. These signals were used as a negative control in our system, representing probes catching no target in the environment. The typical Raman peaks of Cy5 can be observed, including 1151, 1243, 1356, 1446, and 1597 cm^−1^, for aromatic ring stretches [36]. The completely vibrational pattern from the hybridization of Probe-21-Q and miR-21-Cy5 was observed for detection as the Raman spectrum shown in Figure 6A. The signal-to-noise ratio (S/N) was calculated by dividing hybridized miR-21-Cy5 signals to probe-only signals. The S/N ratios at 987, 1356, 1446, and 1597 cm^−1^ were 3.9, 5.2, 12.5, and 6.0, respectively. We further analyzed the LOD for miR-21-Cy5 at 1466 cm^−1^ by 3 times the deviations divided by the slope of calibration. The LODs of these devices were around 0.1 nM, which are compatible with RT-PCR sensitivity. The RSD calculated for 0.1 nM was 11.2%.

Specific analysis for miR-21 was determined as shown in Figure 6C,D. For sequence competitive testing, Probe-21-Q was immobilized and hybridized with miR-21-cy5 on the SiNPs and then competed with miR-21 and miR-21 sequences with 2, 4, and 6 base mismatches. Therefore, the Cy5-related Raman peaks at 1446 cm^−1^ decreased 61% ± 5%, 15% ± 9%, 5% ± 13 %, and 4% ± 17% after competing with miR-21, miR-21-mis2, miR-21-mis4, and miR-21-mis6, respectively.

## 4. Discussion

In this study, high-aspect-ratio SiNPs with corrugated surfaces were designed and fabricated by employing a nanoshielding mechanism [32] with effective SERS enhancement, as shown in Figure 1. To provide different sizes of nanomasks, condensed polystyrene bead monolayers were shrunk from 530 to 450–165 nm in our experiments by applying different RIE times for metal-assisted etching. Due to the nanoscale mask and anisotropic metal-assisted etching process, nanopillar structures were fabricated with high-aspect-ratio pillars. We observed that RIE-160 s was rougher than other cases, but it was not easy to observe roughness in SEM images. We analyzed microrange roughness (R_m_) and nanorange roughness (R_n_) parameters on these surface-corrugated SiNPs separately. R_m_ was analyzed in the 7 μm range area, and R_n_ was analyzed among the pillars that indicated nanoscale roughness changes. In microranges and nanoranges, surface-corrugated SiNPs had a consistently incremental trend with RIE time, but the slope of R_m_ raised faster than that of R_n_. R_n_ was sufficiently increased, which indicated that the number of nanostructures (i.e., hot spots) among the heads of the pillars markedly increased. We further analyzed the correlation between SERS efficiencies and micro/nanorange roughness to investigate their relationship.

We demonstrated the SERS enhancement of the surface-corrugated SiNPs with a common Raman dye, R6G (Figure 4). In our findings, the SERS enhancement relationship was more strongly correlated with R_n_ (0.998) than with R_m_ (0.810). Although R_m_ and R_n_ increases both varied with RIE time, the data suggest that R_n_ dominated much more in SERS enhancements. R_n_ represented the nanocorrugations among the pillars, which confirmed our hypothesis that nanorange roughness dominates the SERS effect. According to the theories of SERS enhancement [43], many studies have proposed the same concepts, namely, that surface roughness [47,48] or a sub-10 nm gap [47] can promote the electric field at hot spots and improve SERS enhancements, which also anchors our suggestion. Therefore, the surface-corrugated SiNPs with RIE-160 s provided around a 1.0 × 10^7^ SERS enhancement, as shown in Figure 5. If we consider the whole device in real applications, the surface-corrugated SiNPs in this study can offer more than 77-fold signal enhancement when compared with an Au-coated silicon substrate with a similar surface area for sensor applications. Although some studies [28,49] using nanoparticles produced higher EFs, their nanostructures might not be easily repeated/maintained in mass production. It should be noted that gold instead of sliver (which might provide an additional 1 order of EF at least) was chosen in this study as a SERS layer for the purpose of long-term material stability.

The detection capability of miR-21-Cy5 using a portable Raman instrument is shown in Figure 6. Raman signals for specific peaks of nucleotides can be clearly visualized at a hybridizing situation for SERS measurement. The detection limit of the SiNPs was also investigated by detecting a 100 pM miRNA-21 sample. The concentration was comparable to the minimum level—10 ng sample (in 2 ng/μL)—collected from human blood for standard RT-PCR enlargement and detection [50,51,52]. Furthermore, the specificity of the SiNPs was evaluated by sequence replacement detection. Figure 6C,D, respectively, show Raman spectra and signal quantification in existing target miR-21-Cy5 and in competition with its mismatched sequences. As an example, the fully matched miR-21 decreased more than 60% of signals, which revealed reasonable results that more than 50% of miR-21-cy5 were replaced with miR-21. In comparison, the 2-mismatched sequences (miR-21-mis2) only slightly decreased their SERS signals, and the 4-mismatches and 6-mismatches did not obviously decrease their SERS signals. These results also correspond with solution-based fluorescent detection (shown in Figure S4). These observations establish that the proposed SiNP sensors achieved good selectivity to 2-base-mismatched miRNA and show great potential in applications for detecting miRNA targets specifically in complex biological samples. Moreover, the detection time can be reduced to less than an hour without the RT-PCR process by employing the proposed nanobiosensor with corrugated nanopillar arrays. We also evaluated the uniformity across the SiNPs for miR-21 detection. According to the analysis, the RSDs were 11.2% for signals at a peak of 1466 cm^−1^, both of which are similar to the surface roughness (10.3%). These results demonstrate the decent uniformity/consistency of the measurement on the SERS active region for miRNA detection. As a result, SiNPs, when implemented with portable Raman spectroscopy, can be considered as portable miRNA sensors, which can be easily transported to remote areas for recognizing CRC-related miRNA and characterizing different onco-miRs.

## 5. Conclusions

A nanobiosensor with surface-enhanced Raman scattering (SERS) capability has been introduced for highly sensitive miRNA detection in this study. The fabricated nanopillar structures can effectively extend hot spot distribution into the third dimension, providing considerable enhancement in Raman signals, more than 3 orders greater than nonoptimized gold-deposited SiNPs. This nanobiosensor can serve as a SERS solid substrate for miRNA-21 recognition in a rapid and reproducible manner, and the target molecule miR-21 can be easily monitored through Raman spectrum variation with a PCR-comparable concentration at 100 pM. The proposed SiNP sensors achieved good selectivity to 2-base-mismatched miRNA. Furthermore, without the sequence amplification process, the whole testing can be completed in an hour. Moreover, due to their easier integration, nanopillar arrays may be employed in microfluidic platforms with lateral flow [24] or automatic RNA sensing [53]. These compact, portable biosensors are also suitable for emerging point-of-care applications such as pesticide poisoning, drug testing, and toxic chemical analysis.

## Supplementary Materials

The following are available online at https://www.mdpi.com/2079-6374/10/11/163/s1, Figure S1: (A) AFM analysis data for (A) microrange and (B) nanorange roughness. Roughness is presented as R_a_ (black), R_q_ (red), and R_v_ (blue) in our AFM software; Figure S2. Raman spectra of R6G on nanopillars (RIE 40–160 s) and bare silicon; Figure S3. (a) UV–Vis absorption spectrum of Probe-21-Q (blue line) and PL spectrum of miR-21-probe (black line). (b) Fluorescent spectra of miRNA-21-Cy5 only (black curve), Probe-21-Q only (green curve), Probe-21-Q plus miRNA-21-Cy5 (red curve), and background signal (blue curve). Noted that green and blue curves overlap in the figure; Figure S4. Fluorescent increasing signals for the competitive testing for target miR-21-Cy5. Probe-21-Q and target miR-21-Cy5 were replaced with miR-21 with 2, 4, and 6 mismatched bases under the same concentration of 100 nM; Table S1: DNA sequences used in this experiment.
