# Supplementary material for: Development of Surface-Enhanced Raman Scattering (SERS)-Based Surface-Corrugated Nanopillars for Biomolecular Detection of Colorectal Cancer
Source: Biosensors (Basel). 2020 Oct 31;10(11):163. doi: 10.3390/bios10110163 (PMC7692079; doi:10.3390/bios10110163)
Supplement: Supplementary file 1 [file biosensors-10-00163-s001.pdf]

Supplementary Information: Development of Surface-Enhanced Raman Scattering (SERS) based Surface-Corrugated Nanopillars for Biomolecular Detection of Colorectal Cancer

Kuan-Hung Chen, Meng-Ju Pan, Zoljargal Jargalsaikhan, Tseren-Onolt Ishdorj and Fan-Gang Tseng

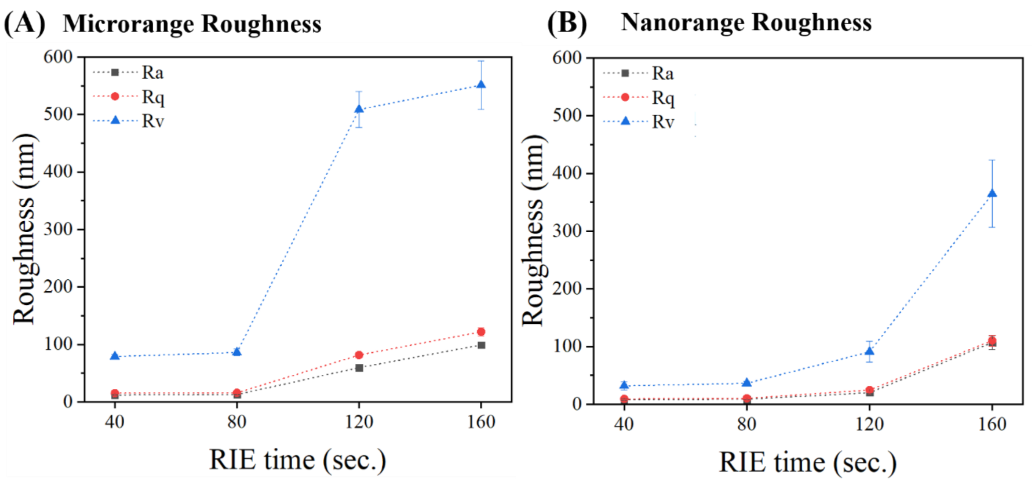

Figure S1. (A) AFM analysis data for (**A**) microrange and (**B**) nanorange roughness. Roughness is presented as R_a (black), R_q (red), and R_v (blue) in our AFM software.

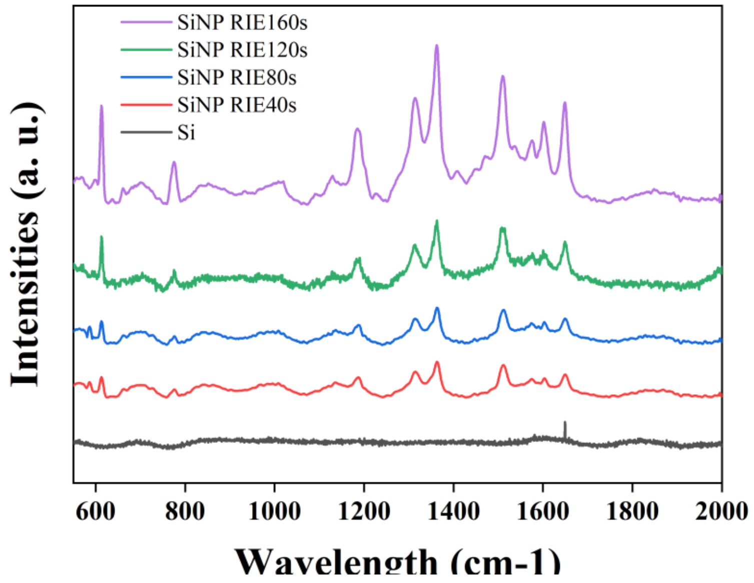

Figure S2. Raman spectra of R6G on nanopillars (RIE 40–160 s) and bare silicon.

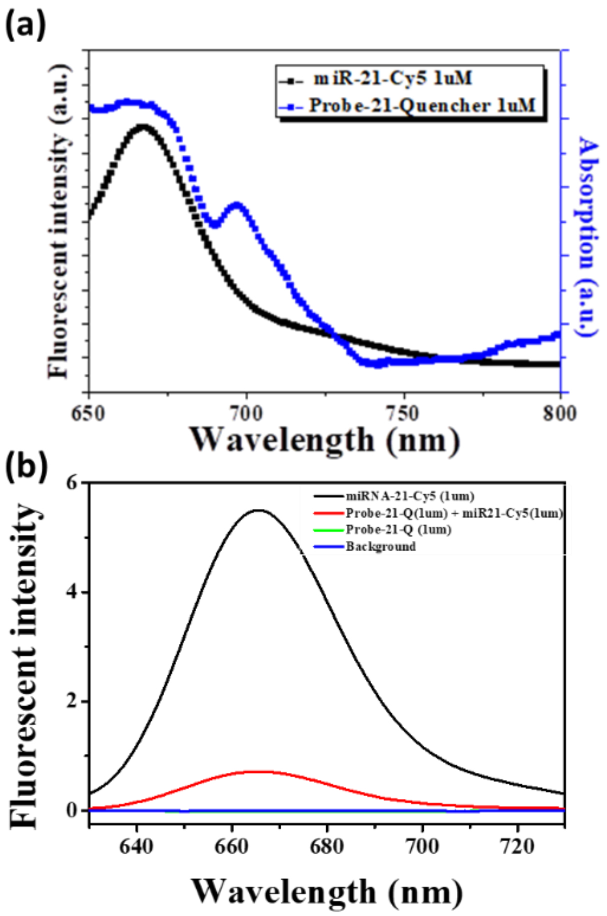

Figure S3. (**a**) UV–Vis absorption spectrum of Probe-21-Q (blue line) and PL spectrum of miR-21-probe (black line). (**b**) Fluorescent spectra of miRNA-21-Cy5 only (black curve), Probe-21-Q only (green curve), Probe-21-Q plus miRNA-21-Cy5 (red curve), and background signal (blue curve). Noted that green and blue curves overlap in the figure.

The photoluminescence spectrum of miR-21-Cy5 was measured by a fluorescence spectrophotometer (FP-6000 series, JASCO, Japan) and is indicated by the black line. The absorption spectrum of Probe-21-Q was measured by a UV–Vis spectrophotometer (UV-670, JASCO, Japan) and is illustrated as the blue line in Figure 6 (a). It can be observed in Figure S3(a) that miR-21-Cy5 has an emission band from 650 to 685 nm and overlaps with Probe-21-Q's strong absorption band (650–740 nm), which is expected in a quenching situation. Figure 6(b) shows the photoluminescence spectra (excitation at 630 nm wavelength) for Probe-21-Q, miR-21-Cy5, and their respective conjugation. Without quenchers, miR-21-Cy5 has an emission peak at 667 nm and Probe-21-Q also has a small emission band at 664 nm. After an hour of incubation, the intensities of miR-21-Cy5 signals decreased in the same manner as Probe-21-Q, which revealed a quenching situation between Probe and targets. These results suggest that Probe-21-Q has an ideal affinity with miR-21-Cy5. Thus, Probe-21-Q was immobilized on the nanopillars for SERS detection of miR-21-Cy5.

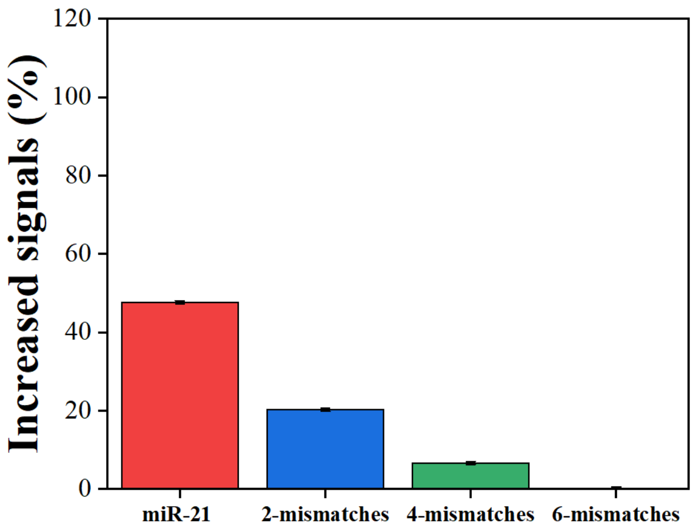

Figure S4. Fluorescent increasing signals for the competitive testing for target miR-21-Cy5. Probe-21-Q and target miR-21-Cy5 were replaced with miR-21 with 2, 4, and 6 mismatched bases under the same concentration of 100 nM.

We demonstrated the specificity of the probe by the fluorescent spectrum. First, miR-21-Cy5 was hybridized with miR-21-Q in PBS solutions. As mentioned in Figure S3, fluorescent signals of miR-21-Cy5 were quenched by miR-21-Q after hybridization, but if miR-21 and other similar sequences replace miR-21-Cy5 from binding sites, the fluorescent signals will increase. miR-21 similar sequences with 2, 4, and 6 mismatched bases replaced the miR-21-Q binding sites under the same concentration of 100 nM. The luminescence signals at 663 nm were in a normalized full-release situation (free miR-21-Cy5) and were compared with fluorescent signals in a fully quenched situation (miR-21-Q hybridized with miR-21-Cy5). After adding miR-21, miR-21-mis2, miR-21-mis2, and miR-21-mis6, the signals increased 47.7%, 20.2%, 6.7%, and 0%, respectively. These indications prove that the probe was sensitive in the two base mismatch recognitions.

Table S1. DNA sequences used in this experiment.

Name	Sequence (5' to 3')
Probe-21-Q	SH-isp18-TCA ACA TCA GTC TGA TAA GCT A-Quencher
miR-21-Cy5	Cy5-TAG CTT ATC AGA CTG ATG TTG A
miR-21	TAG CTT ATC AGA CTG ATG TTG A
miR-21-mis2	TAG CTT ATC AGA CTG ATG CCG A
miR-21-mis4	TAG CTT ATC ACT CTG ATG CCG A
miR-21-mis6	TAG CCC ATC ATC CTG ATG CCG A
